# Consensus‐based follow‐up and treatment registry for *GNAO1* ‐associated disorder

**DOI:** 10.1111/dmcn.70201

**Published:** 2026-02-13

**Authors:** Larissa R. Heideman, Nicole I. Wolf, Moritz Thiel, Jolanda H. Schieving, Chris De Kruiff, Suzanne van den Bos, Eva Van Heeringen‐Broekhuizen, J. Jasper Deuring, Annemieke I. Buizer, Laura A. Van De Pol

**Affiliations:** ^1^ Amsterdam UMC, Vrije Universiteit Amsterdam, Department of Child Neurology Amsterdam Netherlands; ^2^ Amsterdam Neuroscience, Systems & Network Neuroscience Amsterdam Netherlands; ^3^ Amsterdam UMC, Vrije Universiteit Amsterdam, Department of Rehabilitation Medicine Amsterdam Netherlands; ^4^ Amsterdam Movement Sciences, Rehabilitation and Development Amsterdam Netherlands; ^5^ Amsterdam Leukodystrophy Center, Department of Child Neurology, and Amsterdam Neuroscience, Molecular & Genetic Mechanisms Amsterdam University Medical Center, Vrije Universiteit Amsterdam Netherlands; ^6^ Emma Children's Hospital Amsterdam UMC Amsterdam Netherlands; ^7^ Faculty of Medicine and Pediatrics University Hospital Cologne Cologne Germany; ^8^ Department of Pediatric Neurology Radboud University Medical Centre, Amalia Children's Hospital Nijmegen Netherlands; ^9^ Amsterdam UMC, University of Amsterdam, Department of Pediatrics Amsterdam Netherlands; ^10^ Child Neurology, Dutch Epilepsy Institutions Foundation Heemstede Netherlands; ^11^ Stichting GNAO1 NL Leiden Netherlands

## Abstract

**Aim:**

To establish consensus‐based recommendations on relevant domains of functioning and assessment instruments for an *GNAO1*‐associated disorder follow‐up and treatment registry.

**Method:**

This was a mixed‐methods study consisting of a systematic literature search, a survey, and a real‐time Delphi procedure to achieve consensus on domains and instruments. A panel of experts, including health care professionals and individuals with lived experience, was involved in the survey and Delphi procedure. The International Classification of Functioning, Disability and Health (ICF) was used as a framework for the Delphi query.

**Results:**

The Delphi procedure consisted of 67 statements, including 19 on domains and 48 on instruments, identified from the survey and systematic search. The international panel consisted of 27 health care professionals and 10 parents. The panel recommended 17 ICF domains and 26 assessment instruments, mainly in the ‘Body Structures & Functions’ and ‘Activities & Participation’ components.

**Interpretation:**

Using consensus‐based ICF domains and assessment instruments for a *GNAO1* registry is a major step towards harmonization of future international registries, which will facilitate natural history studies and clinical trials.

## Graphical abstract

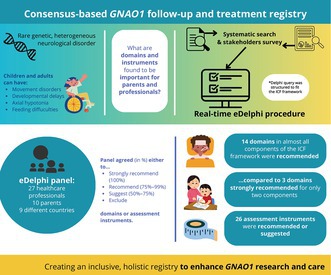

**This original article is commented on by Domínguez‐Carral and Ortigoza‐Escobar on pages** 1182–1183 **of this issue.**

**Abbreviations**
*GNAO1*‐AD: *GNAO1*‐associated disorder
ICF: International Classification of Functioning, Disability and Health
PSI: Parenting Stress Index

**What this paper adds**

- Consensus‐based recommendations for *GNAO1*‐associated disorder (*GNAO1*‐AD) minimize the gap between current research aims and clinical needs.
- Harmonization of international registries is needed for future natural history studies and for the systematic evaluation of treatments for *GNAO1*‐AD.

*GNAO1*‐associated disorder (*GNAO1*‐AD) is a rare genetic neurological disorder typically presenting in childhood. It is caused by heterozygous pathogenic variants in the *GNAO1* gene, which encodes the Go‐alpha subunit of one of the guanine nucleotide‐binding proteins, which have important roles in signal transduction and neurotransmission in the brain.^1^ Currently, 107 pathogenic *GNAO1* variants are known.^2^ Clinical manifestations of *GNAO1*‐AD and its severity vary widely. Typically, *GNAO1*‐AD presents with epilepsy or movement disorders—most often dystonia, chorea, or myoclonus—and developmental delay. An important phenomenon of *GNAO1*‐AD is the susceptibility to develop life‐threatening dyskinetic crises.^3^ The cause or predictors of dyskinetic crises in *GNAO1*‐AD have not yet been identified. In 2023, Sáez González et al.^2^ created an overview of all published genotypes and co‐occurring phenotypes for *GNAO1*‐AD, and demonstrated that even in groups of patients with identical genetic variants, phenotypic heterogeneity exists. Therefore, larger cohort studies are needed to increase our understanding of disease mechanisms, enable better clinical profiling, and ultimately improve personalized treatments.

The rare, severe, and clinically heterogeneous nature of *GNAO1*‐AD poses a great challenge in the care of these patients. To get a better understanding of the natural history, it is necessary to systematically collect longitudinal data on patients with *GNAO1*‐AD, preferably in a multicentre follow‐up registry. To collect relevant data, it is important to identify core domains of functioning and assessment instruments that monitor neurological symptoms and other health‐related domains. These domains and instruments are also essential to evaluate treatment effects on symptoms and disease progression. Ultimately, this could help optimize current therapies, such as deep brain stimulation, and advance emerging clinical trials, such as zinc salt treatment.^4^, ^5^

Therefore, the aim of this study was to identify core domains and assessment instruments for *GNAO1*‐AD using a real‐time Delphi procedure. To explore all health‐related domains relevant to clinicians, parents, and caregivers, we structured this Delphi approach using the International Classification for Functioning, Disability and Health (ICF).^6^ This is a necessary first step in creating a *GNAO1*‐AD registry as part of future natural history studies and for the systematic evaluation of treatments for *GNAO1*‐AD. This study may serve as a basis for clinical guidelines and (inter)national registries.

## METHOD

**Figure 1:**
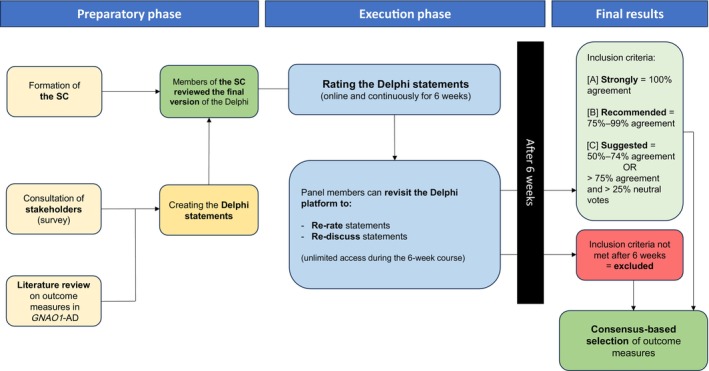
Delphi procedure workflow. Abbreviations: SC, Steering Committee.

To reach consensus on which domains and assessment instruments to include in a *GNAO1*‐AD registry, a modified Delphi procedure was undertaken. A schematic overview of this approach is provided in Figure 1.

The first step of this ‘modified’ approach was to identify assessment instruments for *GNAO1*‐AD previously reported in the literature. A systematic literature search was performed in the PubMed, Cochrane Library, and Embase databases on 3rd November 2023 and was repeated on 21st January 2025. A repeated systematic search was performed after the Delphi procedure was completed to ensure that the literature review remained up to date. To retrieve all studies on *GNAO1*, a broad search string was created, using the term ‘GNAO1’. There were no restrictions for article type, publication status, or language. The title and abstracts of articles on the following inclusion criteria were screened by two reviewers (LRH, TDB): (1) *GNAO1*‐AD; (2) peer‐reviewed studies in human participants; and (3) instruments used to assess the symptoms of *GNAO1*‐AD. Disagreements on study selection were resolved using consensus‐based discussion. Eligible articles were included for full‐text analysis and reported instruments were extracted. Cross‐referencing was performed to identify additional articles.

Simultaneously, a survey among stakeholders was conducted to identify (1) key aspects of *GNAO1*‐AD to monitor and (2) instruments that may not have been described in the literature. Parents of children with *GNAO1*‐AD and health care professionals were invited to participate in a stakeholder survey consisting of five open questions exploring their views on a national *GNAO1*‐AD registry (Appendix S1). All invitees were recruited at the Amsterdam UMC. Participating stakeholders were parents (*n* = 3), (paediatric) neurologists (*n* = 3), paediatric rehabilitation physicians (*n* = 2), a nurse specialized in deep brain stimulation care, a child psychologist, a speech and language therapist, an occupational therapist, and a physiotherapist specialized in the care of children. The answers were qualitatively analysed by one reviewer (LRH).

All collected data from the systematic search and the stakeholder survey were used to construct statements and questions to query the Delphi procedure; these statements and questions were first sent to Steering Committee members to review before the start of the procedure. Members of the Steering Committee consisted of parents (*n* = 2), paediatric neurologists (*n* = 5), a paediatrician, a paediatric rehabilitation physician, and a clinical researcher. The query was structured according to the ICF to cover all health‐related domains using the ICF components (i.e. Body Structures & Functions, Activity & Participation, Environmental Factors & Personal Factors).^6^ Steering Committee members were asked to only give feedback on the structure of the Delphi procedure, and not to provide substantive answers at this stage yet. Next, health care professionals, researchers, parents, and caregivers with expertise in *GNAO1*‐AD or experience in care were invited to participate in the Delphi procedure. Ultimately, the Delphi panel consisted of 37 members from nine different countries (for more details, see Table S1 and Figure S1).

The Delphi procedure was built into a web‐based software (https://www.edelphi.org/), which allows voting participants to edit responses to statements or questions at any given moment, thereby creating a real‐time Delphi procedure independent of traditional voting rounds. Voting and discussion were completed anonymously over the course of 6 weeks between 17th October and 29th November 2024. In the last 3 weeks of the Delphi procedure, ratings (i.e. percentage of votes per statement) were made visible to all panel members. Voting participants were asked to rate statements based on a 5‐point Likert scale. Providing argumentation for a rating was encouraged. Consensus was achieved if the following criteria were met: (1) strongly recommended (100% agreement); (2) recommended (75%–99% agreement); and (3) suggested (50%–74% agreement, or 75% agreement and more than 25% neutral votes).^7^ These levels of agreement were chosen to mirror strength‐of‐recommendation frameworks.^8^ If consensus on a statement was reached after 6 weeks, the statement was included; if not, it was excluded. The progress of the procedure was monitored frequently by one moderator (LRH). Panel members were able to suggest additional key aspects and assessment instruments throughout the whole Delphi procedure. All suggestions were collected at the end of the Delphi procedure and data were qualitatively analysed by the moderator using ATLAS.ti (Scientific Software Development GmbH) for thematic analysis.

The Delphi procedure was moderated by a single researcher (LRH), a physician and researcher with expertise in child neurology. To maintain neutrality, the moderator disclosed her role to the panel at the start of the procedure and documented each step of the qualitative synthesis in an audit trail with original quotations preserved, thus ensuring transparency and prioritizing panellists' perspectives.

## RESULTS

The literature search identified 363 publications of which 260 remained after removal of duplicates before screening. Title and abstract screening led to exclusion of a further 190 articles. Full‐text analysis led to the exclusion of 52 articles. Cross‐referencing identified one article but this was excluded after full‐text analysis because no instrument was reported. A repeated database search resulted in the inclusion of four additional articles, ultimately leading to a total of 22 studies for collection of assessment instruments. An overview of the study selection process can be found in Figure S2; all included studies can be found in Table S2. In total, 17 different instruments were identified. After critical evaluation of relevance by four physician reviewers (LRH, LvdP, NW, AB), this number was reduced to 15 instruments. Two instruments (i.e. electroencephalogram and magnetic resonance imaging) were excluded because they were ancillary investigations. The remaining instruments were integrated into the Delphi query. Two assessment instruments identified in the second literature search were missed in the first literature search (Table S2) and were inadvertently omitted from the Delphi query, that is, the Adaptive Behavior Assessment System and Parenting Stress Index (PSI).

Data from the stakeholder survey was analysed qualitatively by one reviewer (LRH). Key aspects to monitor were identified and grouped into eight categories: (1) treatment; (2) disease‐related factors (i.e. genotype, phenotype, health‐related complications); (3) development; (4) activities of daily living; (5) quality of life; (6) sleep; (7) pain; and (8) care burden. Additionally, 18 assessment instruments were reported in the survey. All categories and instruments were incorporated in the query of the Delphi procedure.

**Figure 2:**
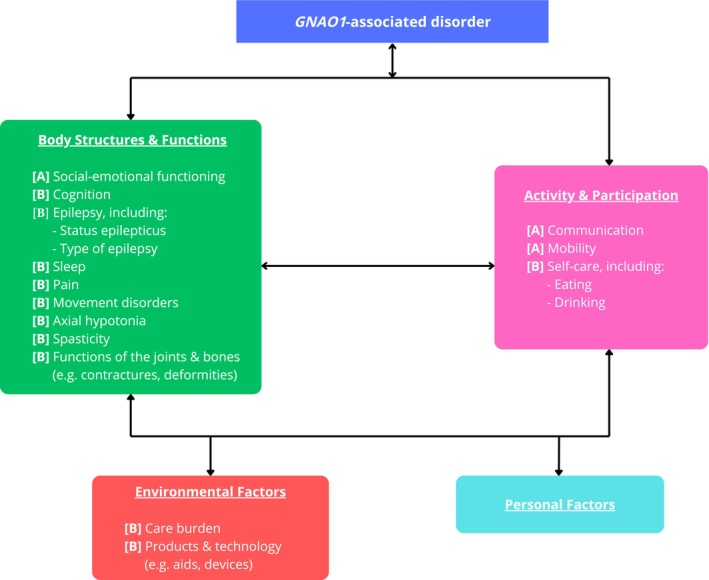
Domains (per ICF component) voted to be included in the registry. The consensus‐based recommendations are levels A (strong recommendation) and B (recommendation).

**Figure 3:**
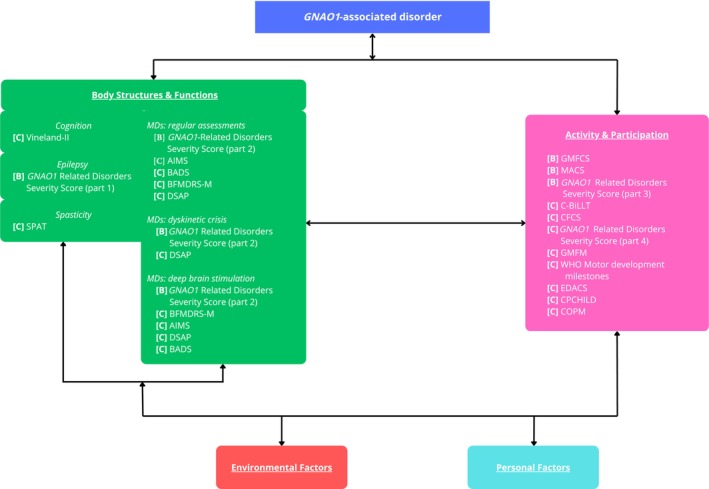
Assessment instruments (per ICF component) voted to be included in the registry. The consensus‐based recommendations are levels B (recommendation) and C (conditional recommendation). Abbreviations: AIMS, Abnormal Involuntary Movements; BADS, Barry–Albright Dystonia Scale; BFMDRS‐M; Burke–Fahn–Marsden Dystonia Rating Scale‐Movement; C‐BiLLT, Computer‐Based Instrument for Low motor Language Testing; CFCS, Communication Function Classification System; COPM, Canadian Occupational Performance Measure; CPCHILD, Caregivers Priorities and Child Health Index of Life with Disabilities; DSAP, Dystonia Severity Action Plan; EDACS, Eating and Drinking Ability Classification System; GMFCS, Gross Motor Function Classification System; GMFM, Gross Motor Function Measure; MACS, Manual Ability Classification System; MD, movement disorder; SPAT, Spasticity Assessment Tool; Vineland‐II, Vineland Adaptive Behavior Scale, Second Edition; WHO, World Health Organization.

**Figure 4:**
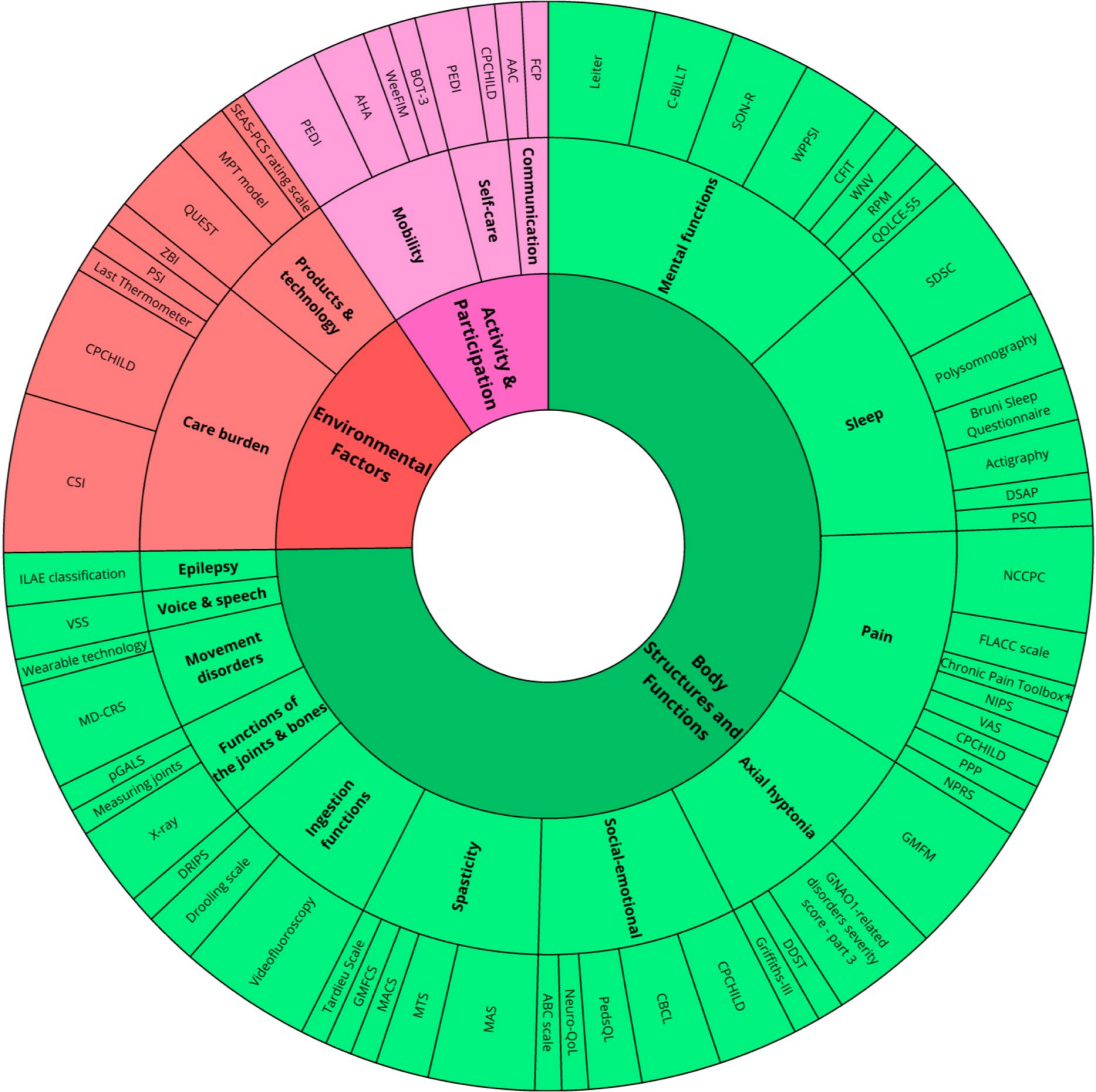
Additional instruments proposed by the panel (no consensus voting). Abbreviations: AAC, Augmentative and Alternative Communication; ABC, Activities‐specific Balance Confidence; AHA, Assisting Hand Assessment; BOT‐3, Bruininks–Oseretsky Test of Motor Proficiency, Third Edition; CBCL, Child Behavior Checklist; CFIT, Cattell Culture Fair Intelligence Test; Chronic Pain Toolbox, Chronic Pain Toolbox for Children with Disabilities; CPCHILD, Caregivers Priorities and Child Health Index of Life with Disabilities; CSI, Caregiver Strain Index; DDST, Denver Developmental Screening Test; DRIPS, Drooling Infants and Preschoolers Survey; DSAP, Dystonia Severity Action Plan; FCP, Functional Communication Profile; FLACC, Faces, Legs, Activity, Cry and Consolability; GMFCS, Gross Motor Function Classification System; GMFM, Gross Motor Function Measure; Griffiths‐III, Griffiths Scales of Child Development, Third Edition; ILAE, International League Against Epilepsy; MACS, Manual Ability Classification System; MAS, Modified Ashworth Scale; MD‐CRS, Movement Disorder Childhood Rating Scale; MPT, Matching Person & Technology; MTS, Modified Tardieu Scale; NCCPC, Non‐communicating Children's Pain Checklist; Neuro‐QoL, Quality of Life in Neurological Disorders; NIPS, Neonatal Infant Pain Scale; NPRS, Numeric Pain Ratings Scale; PEDI, Pediatric Evaluation of Disability; PedsQL, Pediatric Quality of Life Inventory; pGALS, paediatric Gait Arms Legs and Spine; PPP, Paediatric Pain Profile; PSI, Parenting Stress Index; PSQ, Paediatric Sleep Questionnaire; QOLCE‐55, Quality of Life in Childhood Epilepsy Questionnaire‐55; QUEST, Quebec User Evaluation of Satisfaction with assistive Technology; RPM, Ravens Progressive Matrices; SDSC, Sleep Disturbance Scale for Children; SEAS‐PCS, Self‐Reported Experiences of Activity Settings; SON‐R, Snijders‐Oomen niet‐verbale intelligentietests (non‐verbal intelligence tests); VAS, Visual Analog Scale; VSS, Viking Speech Scale; WeeFIM, Functional Independence Measure for Children; WNV, Wechsler Non‐Verbal (Scale of Ability); WPPSI, Wechsler Preschool and Primary Scale of Intelligence; ZBI, Zarit Burden Interview.^15^, ^30^, ^31^, ^32^, ^35^, ^40^, ^41^, ^42^, ^43^, ^44^, ^45^, ^46^, ^47^, ^48^, ^49^, ^50^, ^51^, ^52^, ^53^, ^54^, ^55^, ^56^, ^57^, ^58^, ^59^, ^60^, ^61^

Statements for the Delphi query were created using the categories and assessment instruments identified in the survey and systematic search, and were structured according to the ICF framework. The Delphi query consisted of 67 statements, including 19 on domains and 48 on instruments (Table S3). The panel voted to include all statements on domains and 29 statements on instruments. The domains and instruments voted to be included, derived from the statements, are summarized in Figures 2 and 3, and are structured according to the ICF framework. Two domains with three assessment instruments recommended by the panel did not fit into the ICF framework and are reported separately in this section. Subsequently, 19 statements on instruments were excluded. An overview of all recommended statements with agreement rates can be found in Table S3. Panel members could also propose additional instruments for domains in the comment sections throughout the Delphi procedure. These data were collected at the end of the Delphi procedure. No consensus voting was performed on these data. All proposed instruments have been summarized in Figure 4. All key aspects are summarized in Figures S3 and S4 and Table S4.

To monitor body structures and functions in *GNAO1*‐AD, the Delphi panel strongly recommended one domain, namely social–emotional functioning (level A), and recommended including 10 domains (level B). Four assessment instruments were recommended (level B) and 11 suggested (level C) (Figure 3). For movement disorders, a distinction was made between monitoring symptoms in case of regular assessments or in case of dyskinetic crisis, and whether a child had deep brain stimulation.

To evaluate activity and participation for *GNAO1*‐AD, the panel strongly recommended including communication and mobility (level A), and recommended self‐care, including eating and drinking (level B). For all these domains, three assessment instruments were recommended (level B) and eight suggested (level C), which can be found in Figure 3.

To assess environmental factors for *GNAO1*‐AD, the panel recommended including the following two domains: care burden and products and technology (level B). No assessment instruments were identified in the first systematic search nor the survey; however, the panel proposed five alternative instruments to assess care burden and three to assess products and technology (Figure 4). No consensus voting was performed on these proposed instruments.

To assess personal factors that may affect a patient with *GNAO1*‐AD, no domains or instruments were identified in the first systematic search nor the survey.

Development (level A) and quality of life (level B) were (strongly) recommended by the panel (Table S3). However, as both are overarching concepts, they have not been integrated into the ICF framework in Figure 2. Regarding the assessment instruments, the panel recommended (level B) the Bayley Scales of Infant and Toddler Development, Third Edition for development, and the Child Health Index of Life with Disabilities and Pediatric Quality of Life Inventory for quality of life.

## DISCUSSION

To the best of our knowledge, this is the first study on *GNAO1*‐AD that combines expertise from both health care professionals and individuals with lived experience to determine relevant health‐related domains and assessment instruments for a *GNAO1*‐AD registry. The aim of the registry will be to conduct a longitudinal follow‐up of patients with *GNAO1*‐AD to track the natural history of the disease and evaluate treatments. Identifying appropriate assessment instruments to monitor symptoms remains a challenge in *GNAO1*‐AD because of the heterogeneity of the disease. As shown by our literature search, there is large variability in the instruments used in patients with *GNAO1*‐AD.^5^, ^9^, ^10^, ^11^, ^12^, ^13^, ^14^, ^15^, ^16^, ^17^, ^18^, ^19^, ^20^, ^21^, ^22^, ^23^, ^24^, ^25^, ^26^, ^27^, ^28^, ^29^

In our study, consensus was achieved on 17 domains of functioning and 26 assessment instruments within the ICF framework, for a *GNAO1*‐AD follow‐up and treatment registry; it covers all stages of development. Interestingly, unlike the high level of consensus for domains, consensus on which assessment instruments to use to quantify these domains was considerably lower. This could be because there are instruments identified for the component Body Structures & Functions that have not been validated in children.^30^, ^31^ Moreover, 63 additional instruments, distributed over almost all components of the ICF framework, were proposed by the expert panel. This overview may be useful when additional or alternative instruments are needed.

The domains of communication, mobility, and social–emotional functioning have been strongly recommended by the panel to be included in the registry. In the literature, mobility is well represented,^5^, ^10^, ^15^, ^18^, ^22^, ^27^ but studies on measuring communication skills and social–emotional functioning in patients with *GNAO1*‐AD are scarce. So far, only one study quantified communication in six children with *GNAO1*‐AD by exploring receptive language comprehension using an eye tracking system as a tool to complete conventional tests, and demonstrated that lexical comprehension in three children was normal.^20^ Nonetheless, this experimental paradigm has not yet been validated as an assessment instrument for patients with *GNAO1*‐AD. One of the instruments suggested (level C) was the Computer‐Based Instrument for Low motor Language Testing, which is an instrument developed to assess spoken language comprehension in children with severe motor impairments validated in populations similar to *GNAO1*‐AD.^32^, ^33^ This instrument may be a good alternative to explore language comprehension in children and adults with *GNAO1*‐AD who cannot complete conventional tests because of motor impairments. Measuring social–emotional functioning in *GNAO1*‐AD has been assessed in only one earlier study, which investigated the effects of deep brain stimulation using the Child Health Index of Life with Disabilities.^34^, ^35^ This instrument explores social–emotional functiong in the section called ‘Comfort & Emotions’. The scarcity of research on this domain, relative to the panel's strong recommendation to include social–emotional functioning in a *GNAO1*‐AD registry, illustrates the current misalignment between research aims and clinical needs.

Similarly, for the component ‘Personal Factors’, no domains or assessment instruments were identified in the Delphi procedure. Thus, despite its relevance, this area remains underexplored in *GNAO1*‐AD. These findings are in line with studies reporting on the underrepresentation of ‘Personal Factors’ in other neurodevelopmental disorders.^36^

Notably, care burden was voted in as a level B recommendation (91% agreement). With only one study reporting on an instrument to quantify care burden (using the PSI),^20^ this remains a poorly researched topic. The results of our study show that the PSI indeed may be a useful instrument to fill this gap.^37^ In a paper by Garcia‐Bravo et al., five parents of children with *GNAO1*‐AD were interviewed and described how they experienced transitioning from the sole role of ‘parent’ to caregiver.^38^ In these interviews, the impact of this transition on all aspects of their lives is demonstrated and emphasizes the importance of evaluating care burden on parents and caregivers.

Dominguez‐Carral et al. have developed an instrument to broadly estimate disease severity in *GNAO1*‐AD: the *GNAO1*‐related disorders Severity Score.^15^ In our current study, this instrument has been voted in as an assessment instrument for two components in the ICF framework. This instrument can be useful in clinical care to assess more than just one domain of the ICF framework. However, one major limitation of this assessment instrument is that it only explores certain symptoms of *GNAO1*‐AD. It is not designed to detect subtle changes in specific disease aspects, such as dystonia and epilepsy.

This study has some limitations. First, some assessment instruments included in this Delphi study may be frequently used in clinical practice or research to assess patients with *GNAO1*‐AD but may not be validated for this population or for all age groups. Second, the panel consisted of a large, heterogeneous group of experts, some of whom had specific expertise on particular aspects of *GNAO1*‐AD only. This diversity may have led to skewed agreement rates because certain topics may have been more familiar to some experts than others, potentially affecting the overall consensus. To minimize this effect, there was an option to vote ‘neutral’ on statements in our Delphi query. Nonetheless, this broad representation of expertise also strengthens the validity of these results, having incorporated different perspectives of panellists involved in the care of patients with *GNAO1*‐AD, which can be valuable given the complexity and heterogeneity of this disease. In our study, because of sometimes limited representations of certain subgroups in the panel (e.g. one single physiotherapist), we could not stratify results based on the background of participants. Future studies may consider including more panellists to allow stratification of results and account for differences in expertise. Lastly, there were no additional voting rounds in the Delphi procedure where panel members could also rate newly proposed instruments. This may have led to a smaller number of consensus‐based recommendations. Building on this, two instruments were missed in the first literature search (i.e. the Adaptive Behavior Assessment System and PSI); therefore, panellists could not be addressed in the Delphi query later on. One of these instruments was proposed as an additional instrument by the panel (i.e. PSI) but no consensus voting was performed for it. Although this real‐time Delphi procedure is more time‐efficient for large panels, future studies may take these limitations into consideration when constructing a Delphi procedure.

Having identified core domains of functioning and assessment instruments, the next step is to select a core set of instruments for use in a Dutch *GNAO1*‐AD registry. This selection should be made by a select group of experts to ensure a tailored approach for the Netherlands, as variations may exist across countries. This may be because of limited resources or lack of validated translations of assessment instruments.^39^ The ultimate goal is to select crucial instruments that provide essential information, are accessible for international partners, and remain feasible for routine clinical use. It is crucial that the selection of instruments is made in such a way that alignment with future international registries is ensured because international collaboration is indispensable in research on this ultra‐rare disease.

In conclusion, in this study, consensus‐based recommendations on ICF domains and assessment instruments for a *GNAO1*‐AD registry have been made, which capture the full spectrum of the impact of *GNAO1*‐AD. Our study contributes to personalized care for individuals with *GNAO1*‐AD and is a fundamental first step towards a more unified approach for future research on this ultra‐rare disease.

## Supporting information


**Appendix S1:** Stakeholders survey.

**Figure S1:** Delphi procedure.

**Figure S2:** Overview of the study selection process.

**Figure S3:** Proposed key aspects of functioning for *GNAO1*‐AD.

**Figure S4:** Proposed key aspects of functioning for *GNAO1*‐AD.

**Table S1:** Delphi panel members.

**Table S2:** Included studies retrieved from the systematic literature search.

**Table S3:** Query statements of the Delphi procedure.

**Table S4:** Additional key aspects of functioning (not included: epilepsy and movement disorders).
