# Supplementary material for: Consensus‐based follow‐up and treatment registry for GNAO1 ‐associated disorder
Source: Dev Med Child Neurol. 2026 Feb 13;68(9):1316–24. doi: 10.1111/dmcn.70201 (PMC13436703; doi:10.1111/dmcn.70201)
Supplement: Supplementary file 2 [file DMCN-68-1316-s005.pdf]

Supplementary D: Proposed key aspects of functioning for GNAO1-AD

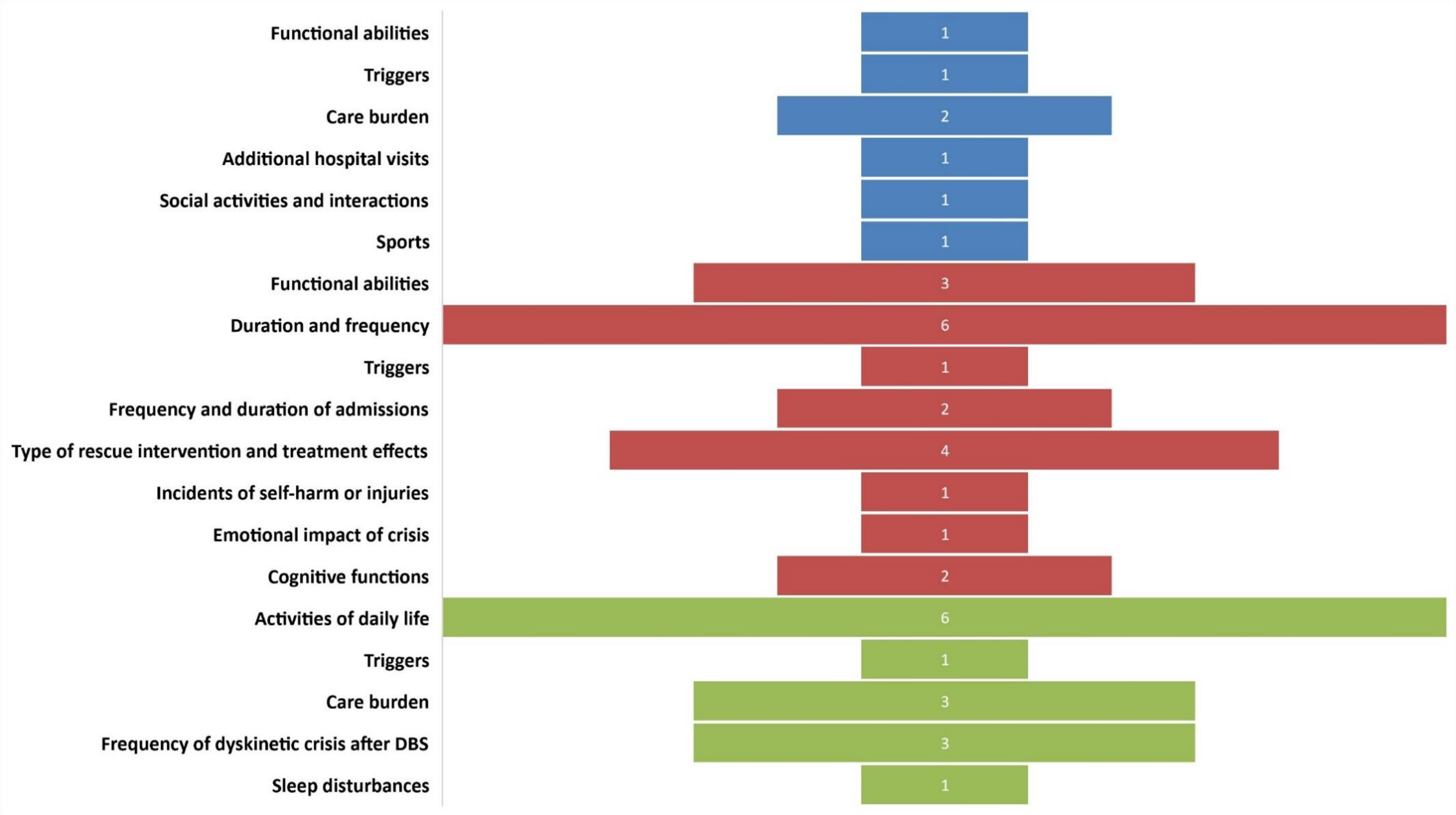

Figure 4. Additional key aspects of functioning, specified for movement disorders (MDs). Numbers in the bars represent the total count a key aspect has been mentioned by panellists. Blue: recommendations for key aspects to monitor MDs; red: in case of dyskinetic crises; green: in presence of deep brain stimulation. DBS = deep brain stimulation.
