# Supplementary material for: Consensus‐based follow‐up and treatment registry for GNAO1 ‐associated disorder
Source: Dev Med Child Neurol. 2026 Feb 13;68(9):1316–24. doi: 10.1111/dmcn.70201 (PMC13436703; doi:10.1111/dmcn.70201)
Supplement: Supplementary file 3 [file DMCN-68-1316-s007.pdf]

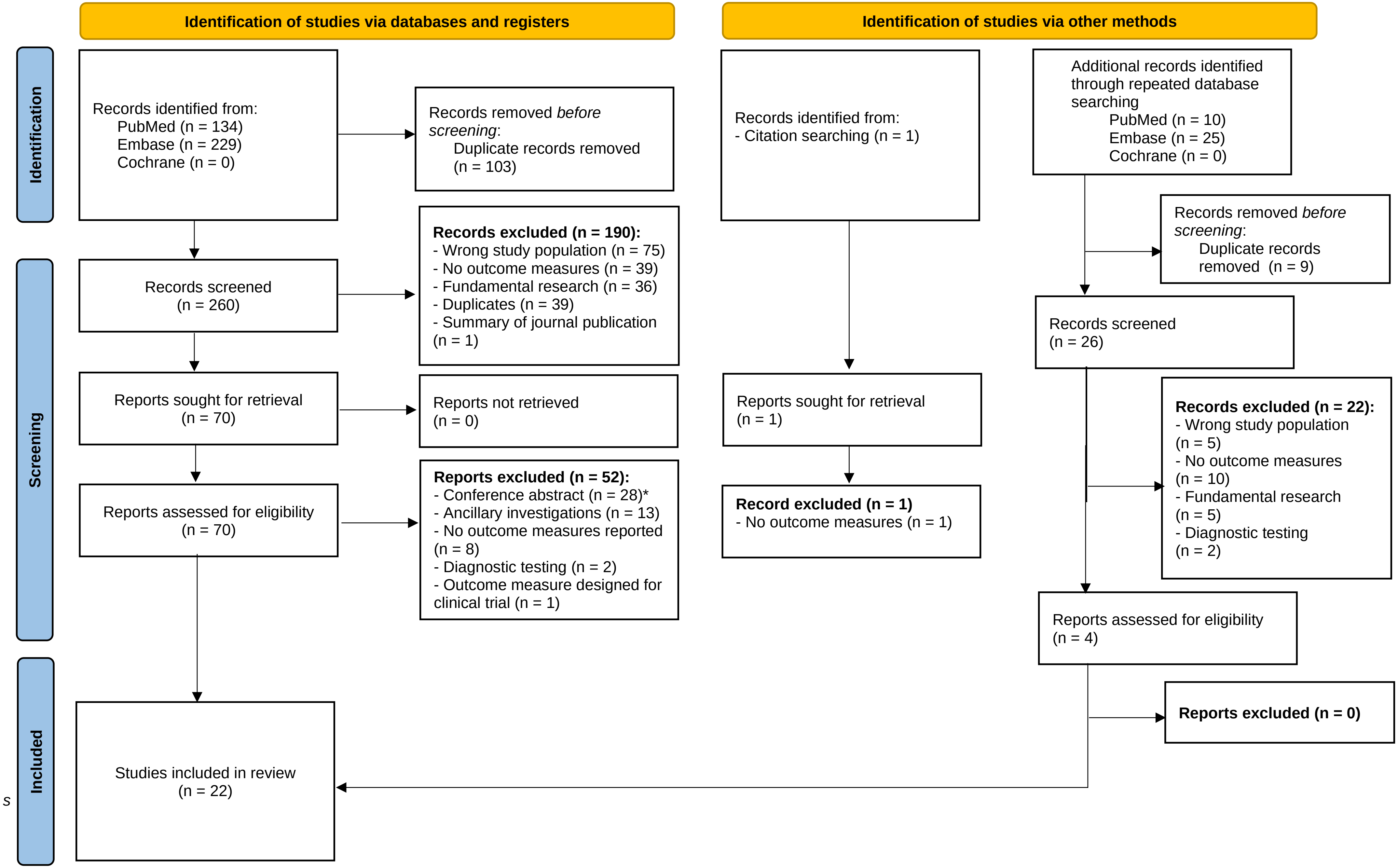

Identification of studies via databases and registers
Identification of studies via other methods
Identification
Screening
Included
Records identified from:
PubMed (n = 134)
Embase (n = 229)
Cochrane (n = 0)
Records removed *before screening*:
Duplicate records removed (n = 103)
Records screened (n = 260)
Records excluded (n = 190):
- Wrong study population (n = 75)
- No outcome measures (n = 39)
- Fundamental research (n = 36)
- Duplicates (n = 39)
- Summary of journal publication (n = 1)
Reports sought for retrieval (n = 70)
Reports not retrieved (n = 0)
Reports assessed for eligibility (n = 70)
Reports excluded (n = 52):
- Conference abstract (n = 28)*
- Ancillary investigations (n = 13)
- No outcome measures reported (n = 8)
- Diagnostic testing (n = 2)
- Outcome measure designed for clinical trial (n = 1)
Studies included in review (n = 22)
Records identified from:
- Citation searching (n = 1)
Additional records identified through repeated database searching
PubMed (n = 10)
Embase (n = 25)
Cochrane (n = 0)
Records removed *before screening*:
Duplicate records removed (n = 9)
Records screened (n = 26)
Records excluded (n = 22):
- Wrong study population (n = 5)
- No outcome measures (n = 10)
- Fundamental research (n = 5)
- Diagnostic testing (n = 2)
Reports sought for retrieval (n = 1)
Record excluded (n = 1)
- No outcome measures (n = 1)
Reports assessed for eligibility (n = 4)
Reports excluded (n = 0)
s
