# Supplementary material for: Consensus‐based follow‐up and treatment registry for GNAO1 ‐associated disorder
Source: Dev Med Child Neurol. 2026 Feb 13;68(9):1316–24. doi: 10.1111/dmcn.70201 (PMC13436703; doi:10.1111/dmcn.70201)
Supplement: Supplementary file 4 [file DMCN-68-1316-s009.pdf]

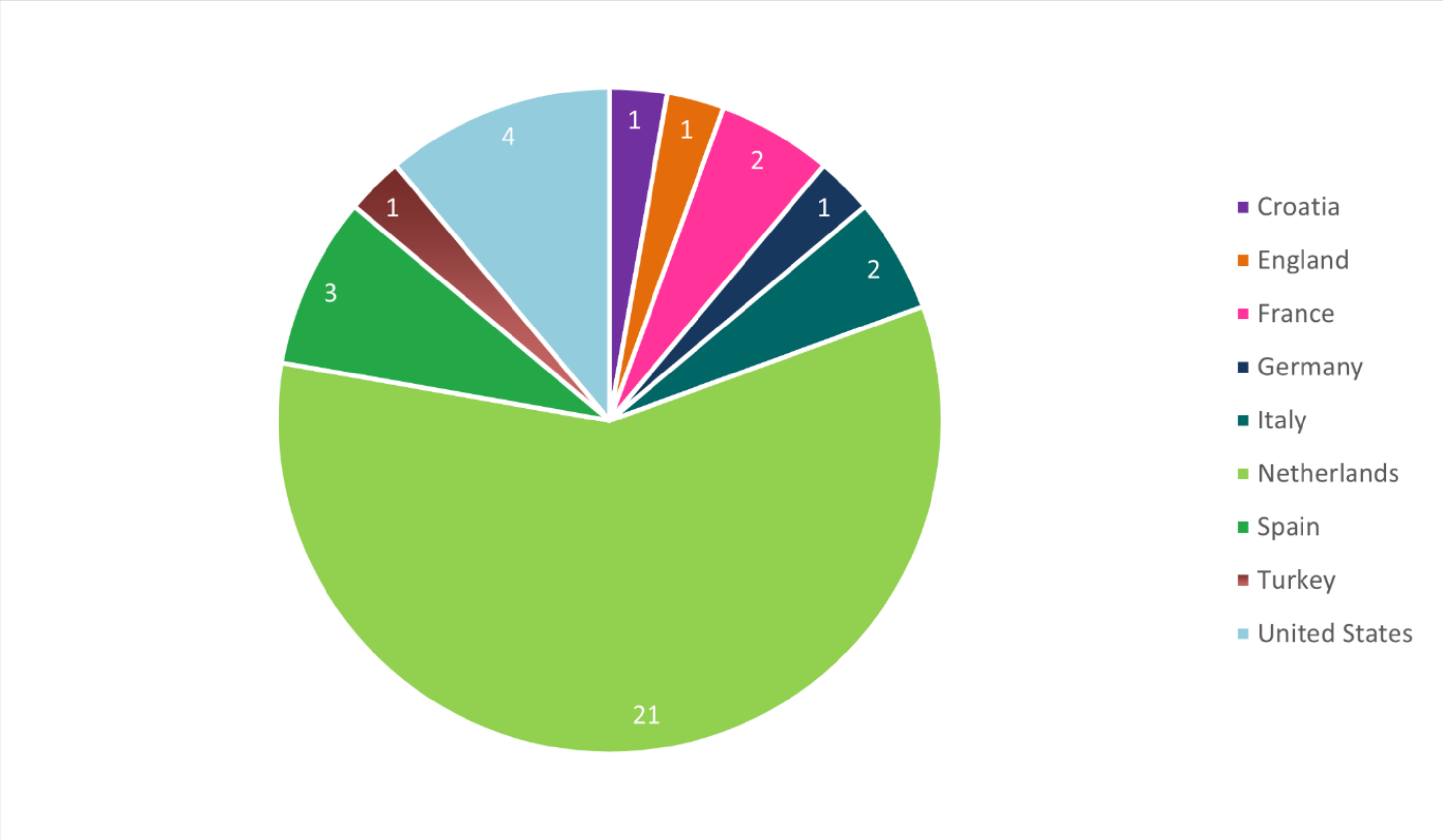

Figure S1. Delphi panel members per country. Here, the total of panellists per country is displayed.
**Panellists from the Netherlands were from five different centres; panellists from the United States were from three different centres.*
