# Supplementary material for: Consensus‐based follow‐up and treatment registry for GNAO1 ‐associated disorder
Source: Dev Med Child Neurol. 2026 Feb 13;68(9):1316–24. doi: 10.1111/dmcn.70201 (PMC13436703; doi:10.1111/dmcn.70201)
Supplement: Supplementary file 5 [file DMCN-68-1316-s002.docx]

**Table S2. Included studies retrieved from the systematic literature search**

| **Author** | **Country** | **Year** | **Aim(s) of study** | **Study design** | **Sample (n=)** | **Age*** | **Sex** | **Genotype** | **Instrument**  **(name)** |
| --- | --- | --- | --- | --- | --- | --- | --- | --- | --- |
| Alkubaisi, A. | Canada | 2022 | To present two cases of DBS for dystonic storm, and to highlight stimulation parameters | Case report + literature review | 2 | 10; 7 | 1 male, 1 female | Not reported | BADS |
| Benato, A. | Italy | 2019 | To report the long-term outcome of four children treated with DBS | Case report + literature review | 2 | 5; 13 | 2 female | c.736G > A, p.Glu246Lys | mMRS |
| Bhardwaj, R. D. | USA | 2015 | To describe the successful use of DBS for symptomatic dystonia | Conference abstract | 1 | 7 | Male | Not reported | BFMDRS |
| Chowdhury, S. | India | 2024 | To highlight a ''milder'' dystonia phenotype of GNAO1 mutation, and its first report from India | Case report | 1 | 18 | Male | c.617G>A, p.Arg206Gln | MMSE |
| Danhofer, P. | Czech | 2021 | To describe patient who underwent DBS | Case report | 1 | 12 | Male | c.625 C > T, p. Arg209Cys | DSAP |
| Domínguez-Carral, J | Spain | 2023 | To develop a severity score for GNAO1-related disorders, and to delineate the correlation between the underlying molecular mechanisms and clinical severity. | Cohort study, methodolo-gical study | 16 | Range: 11M to 15Y | 9 female, 7 male | c.118G>C (p. G40R); c.137A>G (p. K46R); c.143C>T (p.T48I); c.545C>T (p. T182I); c.596T>C (p. L199P); c.626G>C (p. R209P); c.692A>G (p. Y231C); c.704T>C (p. L235P); c.871T>A (p. Y291R); c.607G>C (p.G203R); c.625C>T (p. R209C); c.709G>A (p. E237K) | GNAO1-Related Disorders Severity Score; Vineland-II;  Bayley-III; GMFM-88; BFMDRS; AIMS; |
| Fung, E. L | China | 2022 | To present GNAO1 case treated with DBS | Case report | 1 | 4 | Female | c.709G>A, p.Glu237Lys | BFMDRS |
| Gambar-della, M. L. | Italy | 2023 | To describe the profile of neurovisual competences in children with GNAO1 deficiency to better characterize the phenotype of the disease spectrum | Case series | 7 | 2; 3; 3; 5; 6; 7 | 3 female, 4 male | p.Glu 246Lys, p.Glu 237Lys, p.Glu 246Lys, p.Gly 203Arg, p.Ser 47Gly, p.Gly 203Arg, p.Arg 209Cys | GMFCS |
| Garofalo, M. | Nether-lands | 2023 | To present GNAO1 case treated with DBS | Case report | 1 | 8 | Male | c.626G > A, p.Arg209His | BFMDRS |
| Graziola, F. | Italy | 2021 | To describe their experience in the assessment of the cognitive and communication profile of GNAO1 | Case series | 6 | 15; 3; 4; 8; 7; 3 | 3 female, 3 male | c.709G > A (p. Glu237Lys); c.709G > A (p. Glu237Lys); c.736G > A (p. Glu246Lys); c.625C > T (p. Arg209Cys); c.607G > A (p. Gly203Arg); c.736G > A (p. Glu246Lys) | ABAS; BFMDRS; CFCS; GMFCS; MACS; PedsQL; PSI; RPS; TFL |
| Honey, C. M. | Canada | 2018 | To report a case of GNAO1 with status dystonicus receiving globus pallidus interna (GPi) DBS | Case report | 1 | 10 | 1 male | c. 675G > T, p.Arg209Leu | BADS; CPCHILD |
| Koy, A. | Germany | 2017 | To investigate evolution of symptoms and response to GPi DBS in GNAO1 | Case series | 6 | 9, 14, 14, 11, 7, 10.5 | 2 female, 4 male | c.723+1G>T; c.610G>C (p.Gly204Arg); c.625>T (p.Arg209Cys); c.625>T (p.Arg209Cys); c.709G>A (p.Glu237Lys); c.709G>A (p.Glu237Lys) | BFMDRS-M; BFMDRS-D; GMFCS |
| Lasa-Aranzasti, A. | Spain | 2022 | To present a new case of GNAO1 haploinsufficiency, causing dystonia for which GPi DBS was given | Case report | 1 | 13 | Female | Chr16: 55922943–60313560, GRCh37/hg19 | BFMDRS-M; BFMDRS-D; WISC V test |
| Larasati, Y.A. | Germany | 2025 | Using biochemical, animal, and first-in-human studies, to provide support for the patient stratification and application of zinc acetate in GNAO1-associated disorders | Case study | 1 | 3.4 | Male | c607G>A, p.Gly203Arg | BFMDRS, AIMS, GNAO1 Severity Score, GMFM |
| Levi, V. | Italy | 2024 | To report the safety and efficacy of simultaneous asleep bilateral transcranial magnetic resonance–guided focused ultrasound (MRgFUS) pallidotomy for life-threatening SD | Case report | 1 | 19 | Female | p.Glu246Lys | BFMDRS-M; |
| Liu, Y. | China | 2022 | To present effects of DBS GPi versus subthalamic nucleus (STN) in GNAO1 children | Case series | 3 | 17, 4, 5 | 1 female, 2 male | c.724-8G > A; c.124G > A (p. Gly42Arg); c.124G > A (p. Gly42Arg) | RPM; Gesell developmental schedule; BFMDRS |
| Salazar Villacorta, A. | Spain | 2021 | To evaluate efficiacy and safety of GPi DBS in children with monogenic dystonia | Cohort study | 7 | 13 [9-19] |  | Not reported | BFMDRS-M, BFMDRS-D |
| Thiel, M. | Germany | 2023 | To report on genotype-fenotype of GNAO1 cohort | Cohort study | 25 | Mean: 9 (range: 4M to 24Y) | 12 female, 13 male | c.137A>G (p.Lys46Arg); c.138A>T (p.Lys46Asn); c.170A>C (p.His57Pro); c.218T>A (p.Val73Asp); c.607G>A (p.Gly203Arg); c.607G>A (p.Gly203Arg); c.607G>A (p.Gly203Arg); c.607G>A (p.Gly203Arg); c.607G>A (p.Gly203Arg); c.607G>A (p.Gly203Arg); c.610G>C (p.Gly204Arg); c.625C<T (p.Arg209Cys); c.625C>T (p.Arg209Cys); c.625C>T (p.Arg209Cys); c.626G>A (p.Arg209His); c.674G>A (p.Cyl225Tyr) c.676_677delG1 (p.Val226Argfs*6); c.709G>A (p.Glu237Lys); c.723+1G>T; c.736G>A (p.Glu246Lys); c.736G>A (p.Glu246Lys); c.736G>A (p.Glu246Lys); c.736G>A (p.Glu246Lys); c.863T>C (p.Phe288Ser); c.986del (p.Thr 329Argfs*45) | BFMDRS |
| Wilson, Y.A. | Australia | 2024 | (1) To review the cerebral palsy description/phenotyping of studies reporting on the genetics of cerebral palsy; (2) To report the clinically relevant genes identified in these studies based on our review of the cerebral palsy description/phenotyping. | Systematic review | 15 | 2.9, 7, <19, 12 unreported | 3 males, 3 females, 9 unreported | c.607G >A (p.Gly203Arg); c.626G>T (p.Arg209Leu); c.662C>A (p.Ala221Asp); c.711A>C (p.E237D); c.625C >T (p.Arg209Cys); c.692A>G (p.Tyr231Cys); c.625C>T (p.R209C); c.724-8G>A (c.724-8G>A); c.470T>C (p.L157P); c.626G>A (p.R209H); c.724-8G>A (c.724-8G>A); c.736G>A (p.E246K); c.736G>A (p.E246K); c.736G>A (p.Glu246Lys); c.625C>T (p.Arg209Cys | GMFCS |
| Yamashita, Y. | Japan | 2020 | To report on a novel mutation of GNAO1 | Case report | 1 | 17 | 1 female | c.620C > T (p.S207F) | GMFM; BI |
| Yang, H. | China | 2021 | To report on children receiving DBS for dystonia | Case series | 2 | 4.3; 5.0 | 1 female; 1 male | Not reported | BFMDRS |
| Yilmaz, S. | Turkey | 2016 | To present GNAO1 case treated with DBS | Case report | 1 | 3 | 1 female | NM_020988.2 c.698A > C (p.Q233P) | BFMDRS |

**in years, unless specified otherwise. Adaptive Behavior Assessment Systems (ABAS); Abnormal Involuntary Movement Scale (AIMS); Barry-Albright Dystonia Scale (BADS); The Bayley Scales of Infant and Toddler Development, Third Edition (Bayley-III); Burke-Fahn-Marsden Dystonia Rating Scale (BFMDRS); Disability scale of BFMDRS (BFMDRS-D); Motor scale of BFMDRS (BFMDRS-M); Barthel Index (BI); Communication Classification System (CFCS); Caregivers Priorities and Child Health Index of Life with Disabilities (CPCHILD); Deep brain stimulation (DBS); Dystonia Severity Action Plan (DSAP); Gross Motor Function Classification System (GMFCS); Gross Motor Function Measure (GMFM); Gross Motor Function Measure-88 (GMFM-88); Manual Ability Classification Score (MACS); Mini-Mental State Examination (MMSE); Modified Rankin Scale (MRS); Pediatric Quality of Life Inventory (PedsQL); Parent Stress Inventory (PSI); Raven’s Progressive Matrices (RPS); Italian language test, receptive subtest (TFL); Wechsler Intelligence Scale for Children-V (WISC V Test).*
