# Supplementary material for: Consensus‐based follow‐up and treatment registry for GNAO1 ‐associated disorder
Source: Dev Med Child Neurol. 2026 Feb 13;68(9):1316–24. doi: 10.1111/dmcn.70201 (PMC13436703; doi:10.1111/dmcn.70201)
Supplement: Supplementary file 6 [file DMCN-68-1316-s003.docx]

**Table S1 Delphi panel members**

| **Function** | **Total** |
| --- | --- |
| Parents | 10 |
| Pediatric neurologists | 16 |
| Neurologists with movement disorders expertise | 2 |
| Neurosurgeon | 1 |
| Pediatric rehabilitation physician | 1 |
| Pediatrician | 1 |
| Clinical researcher | 1 |
| Nurse specialised in DBS care | 1 |
| Child psychologist | 1 |
| Speech and language therapist | 1 |
| Occupational therapist | 1 |
| Physiotherapist | 1 |
