# Supplementary material for: Consensus‐based follow‐up and treatment registry for GNAO1 ‐associated disorder
Source: Dev Med Child Neurol. 2026 Feb 13;68(9):1316–24. doi: 10.1111/dmcn.70201 (PMC13436703; doi:10.1111/dmcn.70201)
Supplement: Supplementary file 7 [file DMCN-68-1316-s004.docx]

| **Query category** | **Outcome measure** | **Counts (n)** |
| --- | --- | --- |
| Spasticity | Activities of daily life | 1 |
| Spasticity | Pain | 1 |
| Axial hyptonia | Functional abilities | 1 |
| Axial hyptonia | Use of aids (e.g. orthosis) | 1 |
| Axial hyptonia | Head control | 2 |
| Axial hyptonia | Posture control (incl. degree of support or assistance) | 1 |
| Axial hyptonia | Autonomous sitting | 2 |
| Development | Care burden | 1 |
| Social-emotional | Caregiver reports | 1 |
| Social-emotional | Adapative behavior assessments | 1 |
| Pain | Visual scale (e.g. use of emoticons) | 3 |
| Pain | Comfort scale | 1 |
| Pain | Caregiver reports | 1 |
| Sleep | Caregiver reports (e.g. observations) | 3 |
| Sleep | Visual sleep diaries (e.g. 24-hour sleep-wake chart) | 2 |
| Care burden | Days absent from work | 2 |
| Care burden | Amount of care (e.g. additional caregivers at home) | 1 |
| Care burden | Impact on quality of life | 1 |
| Care burden | Mental health | 1 |

**Table S4. Additional key aspects of functioning (not included: epilepsy and movement disorders)**
