# Supplementary material for: Consensus‐based follow‐up and treatment registry for GNAO1 ‐associated disorder
Source: Dev Med Child Neurol. 2026 Feb 13;68(9):1316–24. doi: 10.1111/dmcn.70201 (PMC13436703; doi:10.1111/dmcn.70201)
Supplement: Supplementary file 8 [file DMCN-68-1316-s006.docx]

**Table S3 Query statements of the Delphi procedure**

| **Number** | **Recommen-dation level** | **Statement*** | **Agree-ment (%)** | Strongly agreed  (%) | *n* | Agreed  (%) | *n* | **Neutral**  **votes (%)** | *n* | **Reponse**  **rate (n)** | **Response**  **rate (%)** |
| --- | --- | --- | --- | --- | --- | --- | --- | --- | --- | --- | --- |
| 1 | **B** | Epilepsy should be checked for regularly in children with GNAO1-related disorder, and therefore added as an outcome measure in a national registry. | **97%** | 71% | 25 | 26% | 9 | **14%** | 5 | 35 | 100% |
| 2 | **B** | The type of epilepsy (epilepsy syndrome) should be determined and monitored over time, and therefore added as an outcome measure in a national registry. | **80%** | 43% | 15 | 37% | 13 | **14%** | 5 | 35 | 100% |
| 3 | **B** | The following measure(s) can be used to monitor and/or describe severity of epilepsy. How useful do you find it to measure epilepsy using the following measure(s): **a. GNAO1-Related Disorders Severity Score (part 1)** | **82%** | 24% | 8 | 59% | 20 | **15%** | 5 | 34 | 97% |
| 4 | **B** | The occurrence of status epilepticus (frequency and duration) in children with GNAO1-related disorder should be monitored and therefore added as an outcome measure in a national registry. | **91%** | 56% | 19 | 35% | 12 | **9%** | 3 | 34 | 97% |
| 5 | **B** | Movement disorders should be monitored and assessed in children with GNAO1-related disorder, and therefore added as an outcome measure in a national registry. | **94%** | 94% | 30 | 0% | 0 | **6%** | 2 | 32 | 91% |
| 6 | **C** | The following measure(s) can be used to monitor and/or score the severity of movement disorders present. How useful do you find it to measure movement disorders using the following measure(s): **a. Abnormal Involuntary Movement Scale (AIMS)** | **68%** | 14% | 4 | 54% | 15 | **21%** | 6 | 28 | 80% |
| 7 | **C** | The following measure(s) can be used to monitor and/or score the severity of movement disorders present. How useful do you find it to measure movement disorders using the following measure(s): **b. Barry-Albright Dystonia Scale (BADS)** | **67%** | 7% | 2 | 59% | 16 | **30%** | 8 | 27 | 77% |
| 8 | **C** | The following measure(s) can be used to monitor and/or score the severity of movement disorders present. How useful do you find it to measure movement disorders using the following measure(s): **c. Burke-Fahn-Marsden Dystonia Rating Scale - Motor (BFMDRS-M)** | **56%** | 36% | 9 | 20% | 5 | **36%** | 9 | 25 | 71% |
| 9 | **C** | The following measure(s) can be used to monitor and/or score the severity of movement disorders present. How useful do you find it to measure movement disorders using the following measure(s): **d. Dystonia Severity Action Plan (DSAP)** | **56%** | 26% | 7 | 30% | 8 | **37%** | 10 | 27 | 77% |
| 10 | **B** | The following measure(s) can be used to monitor and/or score the severity of movement disorders present. How useful do you find it to measure movement disorders using the following measure(s): **e. GNAO1-Related Disorders Severity Score (part 2)** | **86%** | 32% | 9 | 54% | 15 | **14%** | 4 | 28 | 80% |
| 11 | **Excluded** | In case of a hyperkinetic crisis, how useful do you find it to measure severity using the following measure(s): **a. AIMS** | **43%** | 4% | 1 | 39% | 9 | **26%** | 6 | 23 | 66% |
| 12 | **Excluded** | In case of a hyperkinetic crisis, how useful do you find it to measure severity using the following measure(s): **b. BADS** | **22%** | 0% | 0 | 22% | 5 | **35%** | 8 | 23 | 66% |
| 13 | **Excluded** | In case of a hyperkinetic crisis, how useful do you find it to measure severity using the following measure(s): **c. BFMDRS-M** | **30%** | 9% | 2 | 22% | 5 | **26%** | 6 | 23 | 66% |
| 14 | **C** | In case of a hyperkinetic crisis, how useful do you find it to measure severity using the following measure(s): **d. DSAP** | **74%** | 44% | 12 | 30% | 8 | **19%** | 5 | 27 | 77% |
| 15 | **B** | In case of a hyperkinetic crisis, how useful do you find it to measure severity using the following measure(s): **e. GNAO1-Related Disorders Severity Score (part 2)** | **81%** | 35% | 9 | 46% | 12 | **15%** | 4 | 26 | 74% |
| 16 | **C** | In case a child has deep brain stimulation for GNAO1-related movement disorder(s), how useful do you find it to measure symptoms of the movement disorder(s) using the following measure(s): **a. AIMS** | **60%** | 8% | 2 | 52% | 13 | **24%** | 6 | 25 | 71% |
| 17 | **C** | In case a child has deep brain stimulation for GNAO1-related movement disorder(s), how useful do you find it to measure symptoms of the movement disorder(s) using the following measure(s): **b. BADS** | **50%** | 8% | 2 | 42% | 10 | **46%** | 11 | 24 | 69% |
| 18 | **C** | In case a child has deep brain stimulation for GNAO1-related movement disorder(s), how useful do you find it to measure symptoms of the movement disorder(s) using the following measure(s): **c. BFMDRS-M** | **72%** | 28% | 7 | 44% | 11 | **24%** | 6 | 25 | 71% |
| 19 | **C** | In case a child has deep brain stimulation for GNAO1-related movement disorder(s), how useful do you find it to measure symptoms of the movement disorder(s) using the following measure(s): **d. DSAP** | **52%** | 37% | 10 | 15% | 4 | **33%** | 9 | 27 | 77% |
| 20 | **B** | In case a child has deep brain stimulation for GNAO1-related movement disorder(s), how useful do you find it to measure symptoms of the movement disorder(s) using the following measure(s): **e. GNAO1-Related Disorders Severity Score (part 2)** | **89%** | 33% | 9 | 56% | 15 | **7%** | 2 | 27 | 77% |
| 21 | **B** | Spasticity should be monitored and assessed in children with GNAO1-related disorder, and therefore added as an outcome measure in a national registry. | **87%** | 45% | 14 | 42% | 13 | **10%** | 3 | 31 | 89% |
| 22 | **C** | The following measure(s) can be used to monitor and/or score spasticity. How useful do you find it to measure spasticity using the following measure(s): **a. Spasticity Assessment (SPAT)** | **65%** | 6% | 2 | 58% | 18 | **32%** | 10 | 31 | 89% |
| 23 | **B** | Axial hypotonia should be checked for regularly in children with GNAO1-related disorder, and therefore added as an outcome measure in a national registry. | **97%** | 55% | 17 | 42% | 13 | **3%** | 1 | 31 | 89% |
| 24 | **B** | Scoliosis, hip migration or luxation, and contractures of the arms or legs should be monitored and assessed in children with GNAO1-related disorder, and therefore added as an outcome measure in a national registry. | **91%** | 53% | 17 | 38% | 12 | **6%** | 2 | 32 | 91% |
| 25 | **A** | Development should be monitored and assessed in children with GNAO1-related disorder, and therefore added as an outcome measure in a national registry. | **100%** | 82% | 27 | 18% | 6 | **0%** | 0 | 33 | 94% |
| 26 | **B** | The following measure(s) can be used to monitor and/or score development. How useful do you find it to measure development using the following measure(s): **a. Bayley-III** | **83%** | 33% | 10 | 50% | 15 | **10%** | 3 | 30 | 86% |
| 27 | **Excluded** | The following measure(s) can be used to monitor and/or score development. How useful do you find it to measure development using the following measure(s): **b. Gesell Developmental Schedule** | **21%** | 4% | 1 | 18% | 5 | **71%** | 20 | 28 | 80% |
| 28 | **C** | The following measure(s) can be used to monitor and/or score development. How useful do you find it to measure development using the following measure(s): **c. WHO Motor development milestones** | **57%** | 11% | 3 | 46% | 13 | **25%** | 7 | 28 | 80% |
| 29 | **B** | Cognition should be monitored and assessed in children with GNAO1-related disorder, and therefore added as an outcome measure in a national registry. | **97%** | 67% | 22 | 30% | 10 | **3%** | 1 | 33 | 94% |
| 30 | **Excluded** | The following measure(s) can be used to monitor and/or score cognitive functions. How useful do you find it to measure cognition using the following measure(s): **a. Raven’s Progressive Matrices** | **24%** | 0% | 0 | 24% | 7 | **66%** | 19 | 29 | 83% |
| 31 | **C** | The following measure(s) can be used to monitor and/or score cognitive functions. How useful do you find it to measure cognition using the following measure(s): **b. Vineland-II** | **70%** | 17% | 5 | 53% | 16 | **27%** | 8 | 30 | 86% |
| 32 | **Excluded** | The following measure(s) can be used to monitor and/or score cognitive functions. How useful do you find it to measure cognition using the following measure(s): **c. WISC V Test** | **38%** | 0% | 0 | 38% | 11 | **31%** | 9 | 29 | 83% |
| 33 | **Excluded** | The following measure(s) can be used to monitor and/or score cognitive functions. How useful do you find it to measure cognition using the following measure(s): **d. WAIS IV Test** | **22%** | 0% | 0 | 22% | 6 | **44%** | 12 | 27 | 77% |
| 34 | **A** | Communication should be monitored and assessed in children with GNAO1-related disorder, and therefore added as an outcome measure in a national registry. | **100%** | 84% | 27 | 16% | 5 | **0%** | 0 | 32 | 91% |
| 35 | **C** | The following measure(s) can be used to monitor and/or score communication. How useful do you find it to measure communication using the following measure(s): **a. C-BiLLT (Computer-Based Instrument for Low motor Language Testing)** | **69%** | 45% | 13 | 24% | 7 | **31%** | 9 | 29 | 83% |
| 36 | **C** | The following measure(s) can be used to monitor and/or score communication. How useful do you find it to measure communication using the following measure(s): **b. Communication Function Classification System (CFCS)** | **61%** | 32% | 9 | 29% | 8 | **39%** | 11 | 28 | 80% |
| 37 | **Excluded** | The following measure(s) can be used to monitor and/or score communication. How useful do you find it to measure communication using the following measure(s): **c. Communication Matrix** | **26%** | 7% | 2 | 19% | 5 | **74%** | 20 | 27 | 77% |
| 38 | **Excluded** | The following measure(s) can be used to monitor and/or score communication. How useful do you find it to measure communication using the following measure(s): **d. Intelligibility Classification System (ICS)** | **44%** | 7% | 2 | 37% | 10 | **56%** | 15 | 27 | 77% |
| 39 | **Excluded** | The following measure(s) can be used to monitor and/or score communication. How useful do you find it to measure communication using the following measure(s): **e. Nederlands Dysartrie Onderzoek (NDO-K)** | **15%** | 7% | 2 | 7% | 2 | **78%** | 21 | 27 | 77% |
| 40 | **C** | The following measure(s) can be used to monitor and/or score communication. How useful do you find it to measure communication using the following measure(s): **f. GNAO1-Related Disorders Severity Score (part 4)** | **61%** | 18% | 5 | 43% | 12 | **32%** | 9 | 28 | 80% |
| 41 | **A** | Motor function (i.e. fine and gross) should be monitored and assessed in children with GNAO1-related disorder, and therefore added as an outcome measure in a national registry. | **100%** | 84% | 26 | 16% | 5 | **0%** | 0 | 31 | 89% |
| 42 | **B** | The following measure(s) can be used to monitor and/or score motor function. How useful do you find it to measure development using the following measure(s): **a. Gross Motor Function Classification System (GMFCS)** | **76%** | 48% | 14 | 28% | 8 | **17%** | 5 | 29 | 83% |
| 43 | **Excluded** | The following measure(s) can be used to monitor and/or score motor function. How useful do you find it to measure development using the following measure(s): **b.** **Gross Motor Function Classification – Metachromatic leukodystrophy (GMFC-MLD)** | **36%** | 12% | 3 | 24% | 6 | **64%** | 16 | 25 | 71% |
| 44 | **C** | The following measure(s) can be used to monitor and/or score motor function. How useful do you find it to measure development using the following measure(s): **c. Gross Motor Function Measure (GMFM)** | **74%** | 30% | 8 | 44% | 12 | **22%** | 6 | 27 | 77% |
| 45 | **B** | The following measure(s) can be used to monitor and/or score motor function. How useful do you find it to measure development using the following measure(s): **d. Manual Ability Classification Scale (MACS)** | **75%** | 36% | 10 | 39% | 11 | **25%** | 7 | 28 | 80% |
| 46 | **B** | The following measure(s) can be used to monitor and/or score motor function. How useful do you find it to measure development using the following measure(s): **e. GNAO1-Related Disorders Severity Score (part 3)** | **79%** | 29% | 8 | 50% | 14 | **18%** | 5 | 28 | 80% |
| 47 | **A** | Social-emotional functioning should be monitored and assessed in children with GNAO1-related disorder, and therefore added as an outcome measure in a national registry. | **100%** | 55% | 17 | 45% | 14 | **0%** | 0 | 31 | 89% |
| 48 | **Excluded** | The following measure(s) can be used to monitor and/or score social-emotional functioning. How useful do you find it to measure social-emotional functioning using the following measure(s): **a. Strengths and Difficulties Questionnaire (SDQ)** | **45%** | 7% | 2 | 38% | 11 | **52%** | 15 | 29 | 83% |
| 49 | **B** | Quality of life should be monitored and assessed in children with GNAO1-related disorder, and therefore added as an outcome measure in a national registry. | **97%** | 78% | 25 | 19% | 6 | **3%** | 1 | 32 | 91% |
| 50 | **Excluded** | The following measure(s) can be used to monitor and/or score quality of life. How useful do you find it to measure quality of life using the following measure(s): **a. Canadian Occupational Performance Measure (COPM)** | **48%** | 26% | 7 | 22% | 6 | **44%** | 12 | 27 | 77% |
| 51 | **C** | The following measure(s) can be used to monitor and/or score quality of life. How useful do you find it to measure quality of life using the following measure(s): **b. Caregiver Priorities and Child Health Index of Life with Disabilities (CPCHILD)** | **72%** | 38% | 11 | 34% | 10 | **24%** | 7 | 29 | 83% |
| 52 | **C** | The following measure(s) can be used to monitor and/or score quality of life. How useful do you find it to measure quality of life using the following measure(s): **c. Pediatric Quality of Life Inventory (PedsQL)** | **71%** | 18% | 5 | 54% | 15 | **21%** | 6 | 28 | 80% |
| 53 | **B** | Pain should be monitored and assessed in children with GNAO1-related disorder, and therefore added as an outcome measure in a national registry. | **90%** | 37% | 11 | 53% | 16 | **7%** | 2 | 30 | 86% |
| 54 | **B** | Sleep should be monitored and assessed in children with GNAO1-related disorder, and therefore added as an outcome measure in a national registry. | **97%** | 63% | 20 | 34% | 11 | **3%** | 1 | 32 | 91% |
| 55 | **B** | Eating and drinking should be monitored and assessed in children with GNAO1-related disorder, and therefore added as an outcome measure in a national registry. | **97%** | 69% | 22 | 28% | 9 | **0%** | 0 | 32 | 91% |
| 56 | **Excluded** | The following measure(s) can be used to monitor and/or score motor function. How useful do you find it to measure development using the following measure(s): **a. Dysphagia disorder survey (DDS)** | **29%** | 11% | 3 | 18% | 5 | **71%** | 20 | 28 | 80% |
| 57 | **C** | The following measure(s) can be used to monitor and/or score motor function. How useful do you find it to measure development using the following measure(s): **b. Eating and Drinking Ability Classification System (EDACS)** | **62%** | 28% | 8 | 34% | 10 | **38%** | 11 | 29 | 83% |
| 58 | **Excluded** | The following measure(s) can be used to monitor and/or score motor function. How useful do you find it to measure development using the following measure(s): **c. International Dyspaghia Diet Standardisation Initiative (IDDSI)** | **41%** | 17% | 5 | 24% | 7 | **55%** | 16 | 29 | 83% |
| 59 | **B** | Activities of daily life should be monitored and assessed in children with GNAO1-related disorder, and therefore added as an outcome measure in a national registry. | **90%** | 61% | 19 | 29% | 9 | **3%** | 1 | 31 | 89% |
| 60 | **Excluded** | The following measure(s) can be used to monitor and/or score activities of daily life. How useful do you find it to measure quality of life using the following measure(s): **a. AMC Linear Disability Scale (ALDS)** | **8%** | 4% | 1 | 4% | 1 | **73%** | 19 | 26 | 74% |
| 61 | **Excluded** | The following measure(s) can be used to monitor and/or score activities of daily life. How useful do you find it to measure quality of life using the following measure(s): **b. Barthel Index** | **20%** | 4% | 1 | 16% | 4 | **68%** | 17 | 25 | 71% |
| 62 | **Excluded** | The following measure(s) can be used to monitor and/or score activities of daily life. How useful do you find it to measure quality of life using the following measure(s): **c. Burke-Fahn-Marsden Dystonia Rating Scale – Disability (BFMDRS-D)** | **43%** | 7% | 2 | 36% | 10 | **46%** | 13 | 28 | 80% |
| 63 | **C** | The following measure(s) can be used to monitor and/or score activities of daily life. How useful do you find it to measure quality of life using the following measure(s): **d. COPM** | **52%** | 19% | 5 | 33% | 9 | **48%** | 13 | 27 | 77% |
| 64 | **C** | The following measure(s) can be used to monitor and/or score activities of daily life. How useful do you find it to measure quality of life using the following measure(s): **e. CPCHILD** | **69%** | 28% | 8 | 41% | 12 | **31%** | 9 | 29 | 83% |
| 65 | **Excluded** | The following measure(s) can be used to monitor and/or score activities of daily life. How useful do you find it to measure quality of life using the following measure(s): **f. Modified Ranking Scale (MRS)** | **4%** | 4% | 1 | 0% | 0 | **74%** | 17 | 23 | 66% |
| 66 | **B** | Care burden on parents or caregivers should be monitored and assessed, and therefore added as an outcome measure in a national registry. | **91%** | 59% | 19 | 31% | 10 | **9%** | 3 | 32 | 91% |
| 67 | **B** | Use of aids (e.g. wheelchair, walking aid) or devices (e.g. communication books, tablets) should be monitored and assessed, and therefore added as an outcome measure in a national registry. | **84%** | 48% | 15 | 35% | 11 | **13%** | 4 | 31 | 89% |

Statements were matched to corresponding ICF chapters within components of the ICF-framework. For instance: statement 55 (‘’Eating and drinking’’) is categorized under component ‘’Activity & Participation‘’, further subcategorized under ‘’Selfcare’’ where both eating and drinking belong to according to the ICF.

The following statement categories have been renamed to align with the ICF-framework:

- Statement 24 on ‘’Contractures and bone deformities’’ to ‘’Functions of the joints & bones’’
- Statement 34 on ‘’Language abilities’’ to ‘’Communication’’
- Statement 41 on ‘’Motor function’’ to ‘’Mobility’’
- Statement 59 on ‘’Activities of daily life’’ to ‘’Self-care’’
- Statement 67 on ‘’Use of aids or devices’’ to ‘’Products & technology’’

The following statements cover more than just one component within the ICF-framework, and have been removed from the framework and reported separately:

- Statement 25 on ‘’Development’’
- Statement 49 on ‘’Quality of life’’
