# Supplementary material for: Consensus‐based follow‐up and treatment registry for GNAO1 ‐associated disorder
Source: Dev Med Child Neurol. 2026 Feb 13;68(9):1316–24. doi: 10.1111/dmcn.70201 (PMC13436703; doi:10.1111/dmcn.70201)
Supplement: Supplementary file 9 [file DMCN-68-1316-s008.docx]

**Appendix S1: Stakeholders survey**

1. Which aspects of the disease do you consider important to evaluate and include in the national registry?
For parents/caregivers: Please consider both disease-related symptoms and aspects of daily life that are directly or indirectly affected by the symptoms of GNAO1-AD, for both your child and yourself as a parent/caregiver. For example, regarding eating and drinking—does your child experience feeding difficulties, and do you consider it important to evaluate this regularly?

2. Below is a list of aspects of GNAO1-AD that can be evaluated in the context of research:

- **Motor skills** (e.g., walking independently, using a tablet)
- **Cognitive abilities** (e.g., attention span, response to stimuli)
- **Communication** (e.g., speaking)
- **Epilepsy** (e.g., frequency of seizures)
- **Quality of life**
- **Sleep**
- **Pain**

Do you think these aspects should be included in the national registry? Why, and which of these do you consider most important?

3. Are there any other aspects that should not be missing from a national registry for GNAO1-AD that you have not mentioned earlier? You may list as many as you like.

4. For healthcare professionals: Considering the aspects you previously listed, are there any assessment instruments that you believe should be included in the national registry?
(e.g., for communication: an assessment instrument could be the 'CFCS')

5. Do you have any other comments regarding the national registry?
